# A symbiotic gut bacterium enhances *Aedes albopictus* resistance to insecticide

**DOI:** 10.1371/journal.pntd.0010208

**Published:** 2022-03-04

**Authors:** Haiyang Wang, Hongmei Liu, Hui Peng, Yang Wang, Chongxing Zhang, Xiuxia Guo, Haifang Wang, Lijuan Liu, Wenxiang Lv, Peng Cheng, Maoqing Gong

**Affiliations:** Shandong Institute of Parasitic Diseases, Shandong First Medical University & Shandong Academy of Medical Sciences, Jining, China; International Atomic Energy Agency, AUSTRIA

## Abstract

**Background:**

The increasing insecticide resistance of *Aedes albopictus* puts many countries in Asia and Africa, including China, at great risk of a mosquito-borne virus epidemic. To date, a growing number of researches have focused on the relationship between intestinal symbiotic bacteria and their hosts’ resistance to insecticides. This provides a novel aspect to the study of resistant mechanisms.

**Methods/Findings:**

This study reveals significant composition and dynamic changes in the intestinal symbiotic bacteria of *Ae*. *albopictus* between the resistant and susceptible strains based on full-length sequencing technology. The relative abundance of *Serratia oryzae* was significantly higher in the resistance strain than in the susceptible strains; also, the relative abundance of *S*. *oryzae* was significantly higher in deltamethrin-induced *Ae*. *albopictus* than in their counterpart. These suggested that *S*. *oryzae* may be involved in the development of insecticide resistance in *Ae*. *albopictus*. To explore the insecticide resistance mechanism, adult mosquitoes were fed with GFP-tagged *S*. *oryzae*, which resulted in stable bacterial enrichment in the mosquito gut without affecting the normal physiology, longevity, oviposition, and hatching rates of the host. The resistance measurements were made based on bioassays as per the WHO guidelines. The results showed that the survival rate of *S*. *oryzae*-enriched *Ae*. *albopictus* was significantly higher than the untreated mosquitoes, indicating the enhanced resistance of *S*. *oryzae*-enriched *Ae*. *albopictus*. Also, the activities of three metabolic detoxification enzymes in *S*. *oryzae*-enriched mosquitoes were increased to varying degrees. Meanwhile, the activity of extracellular enzymes released by *S*. *oryzae* was measured, but only carboxylesterase activity was detected. HPLC and UHPLC were respectively used to measure deltamethrin residue concentration and metabolite qualitative analysis, showing that the deltamethrin degradation efficiency of *S*. *oryzae* was positively correlated with time and bacterial amount. Deltamethrin was broken down into 1-Oleoyl-2-hydroxy-sn-glycero-3-PE and 2’,2’-Dibromo-2’-deoxyguanosine. Transcriptome analysis revealed that 9 cytochrome P450s, 8 GSTs and 7 CarEs genes were significantly upregulated.

**Conclusions:**

*S*. *oryzae* can be accumulated into adult *Ae*. *albopictus* by artificial feeding, which enhances deltamethrin resistance by inducing the metabolic detoxification genes and autocrine metabolic enzymes. *S*. *oryzae* is vertically transmitted in *Ae*. *albopictus* population. Importantly, *S*. *oryzae* can degrade deltamethrin *in vitro*, and use deltamethrin as the sole carbon source for their growths. Therefore, in the future, *S*. *oryzae* may also be commercially used to break down the residual insecticides in the farmland and lakes to protect the environment.

## Author summary

This work, for the first time, isolated intestinal symbiotic bacteria *S*. *oryzae* from *Ae*. *albopictus* and verified its role in enhancing insecticide resistance *in vivo* and *in vitro*. We show that *S*. *oryzae* enhances deltamethrin resistance by inducing the metabolic detoxification genes and autocrine metabolic enzymes. This work highlights a new aspect of mosquito resistance. Moreover, *S*. *oryzae*’s capacity to high-effectively degrade insecticide *in vitro* may contribute to environmental protection by degrading the residual insecticide in the farmland and lakes.

## Introduction

*Aedes albopictus*, as a vector of multiple viruses, needs serious attention from the public health authorities worldwide. It can transmit dengue, Zika, and yellow fever viruses causing severe hemorrhagic shock and fever in infected mammals including humans [1–3]. Dengue is a prevalent fever in tropical and subtropical countries infecting ~390 million people worldwide every year with serious clinical symptoms in >25% of cases [4]. Furthermore, such infections are rapidly growing with the growing resistance in *Ae*. *albopictus* [5,6]. Therefore, to control mosquito-borne diseases, the management of resistant mechanisms in mosquitoes has become a key research strategy. Previous studies have shown that mosquito insecticide resistance is potentially increasing via four routes: 1) increase in insecticide metabolic capability; 2) mutation of insecticide action sites; 3) behavioral avoidance of insecticide; 4) thickening of mosquito epidermis reducing insecticide penetration [7–9]. Among these, metabolic changes are considered the major resistance-producing route in mosquitoes, which frequently involves upregulation of genes related to metabolic detoxification enzymes such as cytochrome P450 monooxygenase, glutathione S-transferase and carboxylesterase [10–12].

Importantly, symbiotic bacteria in the mosquito gut is suggested to play an important role in insecticide resistance [6,13]. The diversity and abundance of symbiotic bacteria may vary in different mosquito species; for instance, *Ae*. *albopictus* from central Illinois (USA) showed a lower diversity of intestinal symbiotic bacteria compared to local *Anopheles crucians*, *An*. *quadrimaculatus* and *Ae*. *triseriatus* [14]. In addition, intestinal symbiotic bacteria also vary among the same mosquito population due to different living environments, genetic differences, and the host factors such as age [15]. Water from the breeding site can influence the intestinal symbiotic bacteria in the larval stage while eating behavior can affect the gut microbiota of adult mosquitoes [16]. The gut symbiotic bacteria can benefit the host mosquito in several ways, such as improving environmental resistance/adaptability, increasing immunity against pathogenic microorganisms, promoting ingestion and metabolism of exogenous material including nutritional and toxic substances [17–20].

The first evidence of insecticide resistance due to intestinal microbial symbiosis came from the studies in agricultural pests, especially in *Plutella xylostella* [21,22]. Studies have shown that bacteria such as *Burkholderia mallei*, *Stenotrophomonas maltophilia*, and *Citrobacter amalonaticus* can increase host resistance to insecticides [23–26]. Although it is a well-accepted hypothesis that the mosquito intestinal symbiotic bacteria may enhance the host’s metabolism of insecticides via co-metabolism and/or mineralization [27], it is yet not fully validated; most information has been from species translation studies. Studies showed that *Culex pipiens pallens* and *Ae*. *albopictus* have significant differences in the diversity and abundance of intestinal symbiotic bacteria between the sensitive and resistant strains [6,28]. However, the role of these differences in the development of insecticidal resistance lacks strong evidence. Also, the resistance mechanism involving the main bacteria in *Ae*. *albopictus* remains elusive.

In this study, either laboratory-bred or field-caught deltamethrin-resistant and -susceptible strains of *Ae*. *albopictus* were used to examine the differences in host gut bacterial flora. We showed that bacterial diversity and abundance were significantly higher in resistant strains than in susceptible strains. Furthermore, *S*. *oryzae* was the dominant gut bacteria that increased resistance to deltamethrin by upregulating the metabolic detoxification genes and the secretion of carboxylesterase. This study reveals the mechanism of insecticide resistance in mosquitoes involving intestinal symbiotic bacteria.

## Materials and methods

### Mosquitoes rearing

The *Ae*. *albopictus* susceptible strain, donated by Shandong Provincial Center for Disease Control and Prevention, was bred in Shandong Institute of Parasitic Diseases for >20 years without exposure to any kinds of insecticides. Meanwhile, the insecticide-resistant strain was screened from each generation larval stage (the third or fourth instar) using deltamethrin at the lethal concentration of 50% (LC_50_), and the survivors after screening were used for breeding the next generation. After >3 years of screening, the 52^nd^ generation mosquitoes, whose resistance ratio was 109.6 compared to the susceptible mosquitoes, were considered as resistant strain. Field mosquitoes were caught in August 2019 (Zhenjiang city, Jiangsu province) and May 2020 (Jiaxiang county and Rencheng District, Shandong province) and stably reared in the laboratory. The breeding conditions were as follows: temperature, 26°C ± 2; relative humidity, 75% ± 5; photoperiod, 12:12 hr (light: dark). The larvae were fed with a mixture (1:3) of pork liver powder and yeast powder. Adult mosquitoes were transferred to the mosquito cages and fed with a 10% glucose solution and defibrinated sheep blood through a Hemotek unit. The bioassay to test insecticide resistance of the larva was performed following the World Health Organization (WHO) guidelines: Resistance ratio (RR) = resistant strain LC_50_/susceptible strain LC_50_; the LC_50_ of deltamethrin for each strain is listed in **Table 1** to show their resistant and susceptible status with the fold change. The bioassay for adult mosquitoes was conducted using the CDC bottle method with 0.03% deltamethrin film.

**Table 1:** Sample information of five strains mosquitoes.

	S	R	ZJ	RC	JX
LC_50_ (μg/L)	2	219.2	38.03	69.08	117.6
Resistance ratio	1	109.6	19.02	34.54	58.8
Mortality rate (%)	-	-	66.7±8.3	28.0±12.0	5.3±6.1
Phenotypic indication	susceptible	resistance	susceptible	incipient resistance	resistance

S: laboratory-bred deltamethrin-sensitive mosquitoes, R: laboratory-bred deltamethrin-resistant mosquitoes, ZJ: field mosquitoes caught in Zhenjing, Jiangsu province, RC and JX: field mosquitoes caught in Rencheng and Jiaxiang county, Shandong province. The mortality rate was recorded after the larva of three strains of field-caught mosquitoes were treated 24h by 50 μg/L deltamethrin. “-” means not applicable.

### Full-length 16S rRNA sequencing and analysis of microbial diversity

The surface of adult mosquitoes was first disinfected with 75% alcohol for 3 minutes and then rinsed with sterile 1× PBS buffer for 2 minutes. For each strain, five biological replicates (n = 20 mosquito guts) were set. The intestinal sample tissues were crushed by ultrasonication and the total DNA was extracted following the instructions of the DNA Isolation Kit (AG21007, Accurate Biotech (Hunan) Co., Ltd.). The full-length of 16S rRNA gene was amplified with primer 27F/1492R (**S1 Table**). The PCR reactions were set in 10 μL reaction system with following conditions: 95°C for 5mins; 95°C for 30s; 50°C for 30s; 72°C for 1min, 30 cycles. The PCR products were purified, quantified, and homogenized to create a sequencing library (SMRT Bell). After the quality inspection, a single-molecule sequencer PacBio Sequel was used for library sequencing. UCLUST [29] in the QIIME software (version 1.8.0) was used to cluster tags at a similarity level of 97% and OTUs (Operational taxonomic units) were obtained [30]. The taxonomic annotation of OTUs was performed using the Silva (http://www.arb-silva.de) database.

The detailed methods for bacterial diversity analysis have been earlier described by Wang et al. [6]. The sequencing data have been deposited in the Short Read Archive (NCBI) with an accession number PRJNA687827. [16].

### Screening of main bacteria involved in increased resistance

In order to screen the main bacteria that may promote deltamethrin resistance, we performed a differential screening on the intestinal symbiotic bacteria from different strains of *Ae*. *albopictus* with three different aspects. The comparisons were performed between laboratory resistant (R) and susceptible (S) strains, field-caught resistant mosquitoes from Jiaxiang County (JX mosquitoes) and urban-caught susceptible mosquitoes from Zhenjiang city and Rencheng district (ZJ and RC mosquitoes), and mosquitoes before and after deltamethrin treatment.

The larvae of ZJ mosquitoes were treated with deltamethrin at LC_50_ (38.03μg/L) to observe the changes in the composition and diversity of symbiotic bacteria under the insecticide pressure. Over 200 normally active larvae were treated with deltamethrin, while the control group was fed routinely. After 24h, normally active larvae were selected from both groups as the test samples. Each group had 5 biological replicates. The extraction of 16S rRNA, sequencing, and analysis were performed as above.

### Isolation and culture of *S*. *oryzae*

Adult mosquitoes’ guts were isolated and crushed in PBS with vortexing to fully release the intestinal flora into the buffer. Serially diluted homogenates (10μL) were cultured on the LB medium overnight at 30°C. Based on macroscopic observation, bacteria with obvious differences in colony morphology, transparency, size, luster, and color were delineated on LB medium; in total, thirteen strains (single pure culture) were obtained. The full-length 16S rRNA of these were amplified as described above. The sequencing data were subject to blast in NCBI with GCA_001976145.1 [31] and the colony of *S*. *oryzae* was identified. The isolated colonies were stained with gram and capsular spore stains, and the morphological characteristics were examined using immersion oil microscopy.

### Generation of GFP-tagged *S*. *oryzae* and accumulation in the gut

In order to confirm that *S*. *oryzae* can be accumulated in the intestinal tract of *Ae*. *albopictus* by artificial feeding, eGFP*-*expressing *S*. *oryzae* was constructed for artificial feeding. Plasmid pET28-sfGFP (purchased from Hunan Fenghui Biotechnology Co., Ltd.) simultaneously expresses Green fluorescent protein and kanamycin resistance (50μg/mL). These were transformed into the competent *S*. *oryzae* using the CaCl_2_ chemical transfer method following the instruction of the TaKaRa kit (Code No. 9052).

GFP-tagged *S*. *oryzae* were cultured in LB broth with 50μg/mL kanamycin at 180rpm and 30°C overnight. The cultures were centrifuged at 3000g for 10mins, and the obtained bacterial pellets were washed with sterile PBS thrice using repeated centrifugation and resuspension. Finally, the bacteria were mixed with a 5% sterile glucose solution to obtain a solution of GFP-tagged *S*. *oryzae in* glucose water (hereinafter referred to as "glucose-bacteria solution” (GBS)); the OD_600_ was adjusted to 0.5–0.8. Adult mosquitoes (one day after eclosion in the starving state) were continuously fed with sterile sponges dipped in an appropriate amount of GBS for 48h. The sponges were replaced every 4-6h to reduce the bacterial pollution from the air. Finally, GBS was replaced with a 10% sterile glucose solution, and the feeding continued for 24h; while the control group was always fed with 10% sterile glucose solution. Successively, the intestinal tissues were dissected from each group and placed on a slide containing 10μL PBS to observe the green fluorescence under a fluorescence microscope.

### Preparation of *S*. *oryzae*-enriched and aseptic *Ae*. *albopictus*

A mixture of gentamicin (150μg/mL) and streptomycin (150μg/mL) was mixed with the 5% sterile glucose solution to form a glucose-antibiotic solution (hereinafter referred to as "Sterilization solution"). The feeding was performed as described above, and the control group was fed with a 10% sterile glucose solution.

To validate the antibiotic removal effect on intestinal symbiotic bacteria, two methods, bacterial culture, and quantitation of absolute 16S rRNA copies number were used [32]. In the bacterial culture method, the intestinal tissue homogenates were diluted 10-fold to 10^−4^ in a gradient manner; then, 10μL of each dilution was cultured on LB plates at 30°C for 48 h. Both the experimental and control groups were coated with 3 media to reduce the error from different coating techniques. Finally, the number of respective colonies on the media was counted to calculate CFU/mL. The experiment was repeated twice. For the 16S rRNA copies number estimation, the accustandard (synthesized by Haotian Biotech Co., Ltd.) with known copy number was serially diluted (10^−1^ step) to 10^−5^, and. Then, the DNA of each sample and the accustandard of different concentrations were used as the template for RT-qPCR reaction using 515F and 907R primers. Finally, Ct values were collected to obtain copy number/μL DNA of each sample, and the results were converted into copy number/mosquito.

The *S*. *oryzae*-enriched *Ae*. *albopictus* were prepared according to “section 2.6” and used for further experiments. The susceptible mosquitoes were set as samples from both groups.

### The effect of *S*. *oryzae* enrichment in the intestinal tract of *Ae*. *albopictus*

The aforementioned *S*. *oryzae*-enriched *Ae*. *albopictus* were fed with sterile fibrin-free sheep blood by a Hemotek unit. The engorged and non-engorged mosquitoes were counted; the engorged mosquitoes were placed into individual mosquito cages (10 biological replicates) and fed with a 5% sterile glucose solution. The conventionally reared mosquitoes were used as the control group. The number of eggs and hatching rate during the mosquito life cycle were observed and recorded daily.

### *S*. *oryzae* contribution to the resistance of *Ae*. *albopictus*

*S*. *oryzae*-enriched and untreated ZJ mosquitoes were respectively set as experimental and control groups. Each bottle had no less than 25 vibrant mosquitoes. The blank control group contained acetone but without a deltamethrin film (**S1 Fig**). The mosquitoes were fully exposed to the film for 60 min, and the number of knockdown mosquitoes was recorded every 10 minutes. The knockdown mosquitoes were separately transferred to the recovery bottle after 60 min and fed with a 10% glucose solution to record the survival number after 24 h. The same method was applied for the bioassay of three field-caught mosquito strains. The experiment was repeated twice.

### ELISA to estimate the activities of three main metabolic detoxification enzymes

The activities of cytochrome monooxygenase P450s (CYPs), glutathione -S- transferase (GSTs), and carboxylesterase (CarEs) were determined. The two experimental groups belonging to *S*. *oryzae*-enriched and sterile mosquitoes were set. Also, a control group of conventionally reared adult mosquitoes was set. ZJ mosquitoes were used as samples from the abovementioned three groups. Twenty female mosquitoes from each group were crushed into 300μL PBS buffer, thoroughly mixed, and centrifuged at 5000g for 10mins. The obtained supernatants were subjected to ELISA. The respective ELISA Kits were purchased from Jianglai Biotech. The absorbance of the samples was measured at 450nm by an ELISA reader to calculate the corresponding international units of enzyme activity.

To determine the production/release of the three detoxifying enzymes from bacteria, *S*. *oryzae* was cultured in LB medium at 30°C and 180rpm for 24h. The cultures were centrifuged at 4°C and 10000g for 10mins, and the obtained supernatant was tested for detoxifying enzymes.

### *S*. *oryzae* degrade deltamethrin *in vitro*

*S*. *oryzae* was cultured to OD_600_ = 0.5, 1, and 2, respectively. Bacterial deposits were washed twice with PBS and then resuspended into a 40mL liquid inorganic salt medium. The liquid medium contained deltamethrin as the sole carbon source with an initial concentration of 0.1mg/L. The mixture was incubated at 30°C and 180rpm. 1 mL sample mixture was taken out at 0, 12, 24, 36, and 48h respectively, for Gas Chromatography-Mass Spectrometry (GC-MS/MS) to determine the concentration of deltamethrin and its degradation product. The un-inoculated inorganic salt medium was used as blank control. In this part, a GC-MS analysis platform (Thermo, Ultimate 3000LC, Q Exactive HF) and C18 chromatographic column (Zorbax Eclipse C18(1.8μm*2.1*100mm)) were used. Chromatographic separation conditions were as follows: column temperature, 30°C; flow velocity, 0.3mL/min; mobile phase composition, double distilled water, 0.1% formic acid, chromatographic pure acetonitrile; injection volume, 2 μL; automatic injection temperature, 4°C.

### Differentially expressed genes (RNA- Seq) and RT-qPCR validation

To investigate whether *S*. *oryzae* enhances *Ae*. *albopictus* resistance by inducing the expression of metabolic detoxification-related genes, we examined the transcriptomic data from before and after *S*. *oryzae* enrichment mosquitoes. Two strains *i*.*e*., resistant and Zhenjiang mosquitoes that respectively represent laboratory and field strains were simultaneously sampled and sequenced. Each group contained 20 mosquitoes. Total RNA was extracted by Qiagen RNeasy Mini Kit. The high qualities clean reads were obtained from raw reads after data filtering, which were then compared with the reference genome of Foshan *Ae*. *albopictus* from the Vectorbase database (http://vectorbase.org/vectorbase/app/record/organism/TMPTX_aalbFoshan). The detoxification-related genes with criteria log2 fold change >1 were selected for further analysis. The sequencing data have been deposited in the Short Read Archive (NCBI) with an accession number PRJNA755849.

The mRNA was reverse transcribed to cDNA following the protocol of PrimeScript RT Reagent Kit, which was then used as the template for RT-qPCR reaction. The primers corresponding to 24 differentially expressed genes (DEGs) were designed (listed in **S1 Table**), and the *β*-actin gene was used as the reference gene. RT-qPCR was performed according to the reaction system. Relative quantitative ΔCt and 2^-ΔΔCt^ were used to calculate the differential multiples.

### Transmission mode of *S*. *oryzae*

To test the transmission mode, GFP-tagged *S*. *oryzae* was introduced into engorged adult female mosquitoes by feeding with GBS. Each female mosquito was bred in an individual cage in isolation. Five repetitive simultaneous controls were set, and the respective eggs were collected into separate Ep tubes with 200μL 1×PBS. The eggs were observed under a fluorescence microscope.

To test whether the larvae can directly acquire *S*. *oryzae* from the aquatic breeding sites, water was collected from the respective breeding sites (Zhenjiang, Jiaxiang, Rencheng). **S2 Fig** shows the experimental process. Large water samples (500mL) were used to prevent the false negative of 16S rRNA detection. The water samples were filtered using the sterile filter membrane (0.15μm) and the collected larvae were brought back to the laboratory for the extraction of 16S rRNA and microflora species analysis (as described in “section 2.3”).

### Statistical analysis

CDC bottle bioassay knockdown rate was analyzed by Log-rank (Mental-Cox) test [33]. The correlation between the mosquito survival rate and the relative richness of *S*. *oryzae* was tested by the Pearson correlation analysis. The ability of *S*. *oryzae* to degrade deltamethrin *in vitro* was evaluated using multiple linear regression. The effect of *S*. *oryzae* enrichment on the survival rate was analyzed using Fisher’s exact test. The *t*-test was used for other statistical analyses. All analyses were performed using the GraphPad Prism or SPSS 25 statistical software and *p* = 0.05 was considered statistically significant.

## Results

### Analysis of bacterial diversity

**Fig 1:**
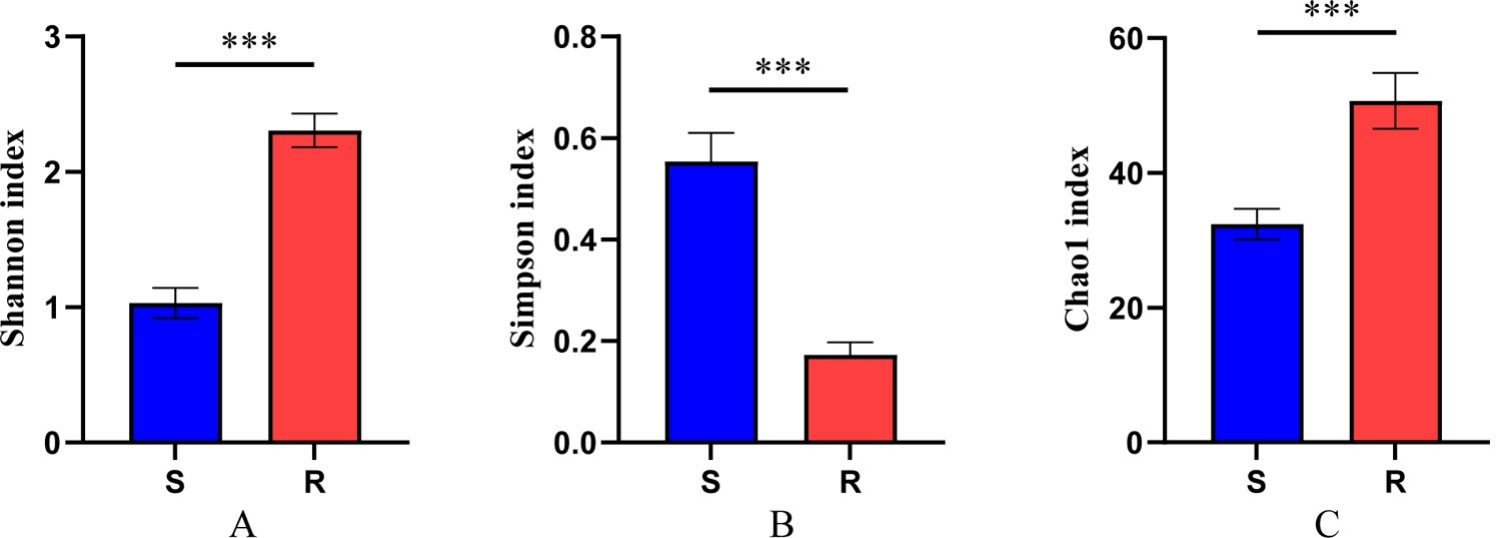
Fig 1 shows the Shannon (A), Simpson (B) (the Simpson index is inversely proportional to diversity) and Chao1 index (C), respectively. S represents sensitive mosquitoes and R represents resistant mosquitoes, indicating that both bacterial diversity and abundance index of resistant mosquitoes significantly higher than that of sensitive mosquitoes.

**Fig 2:**
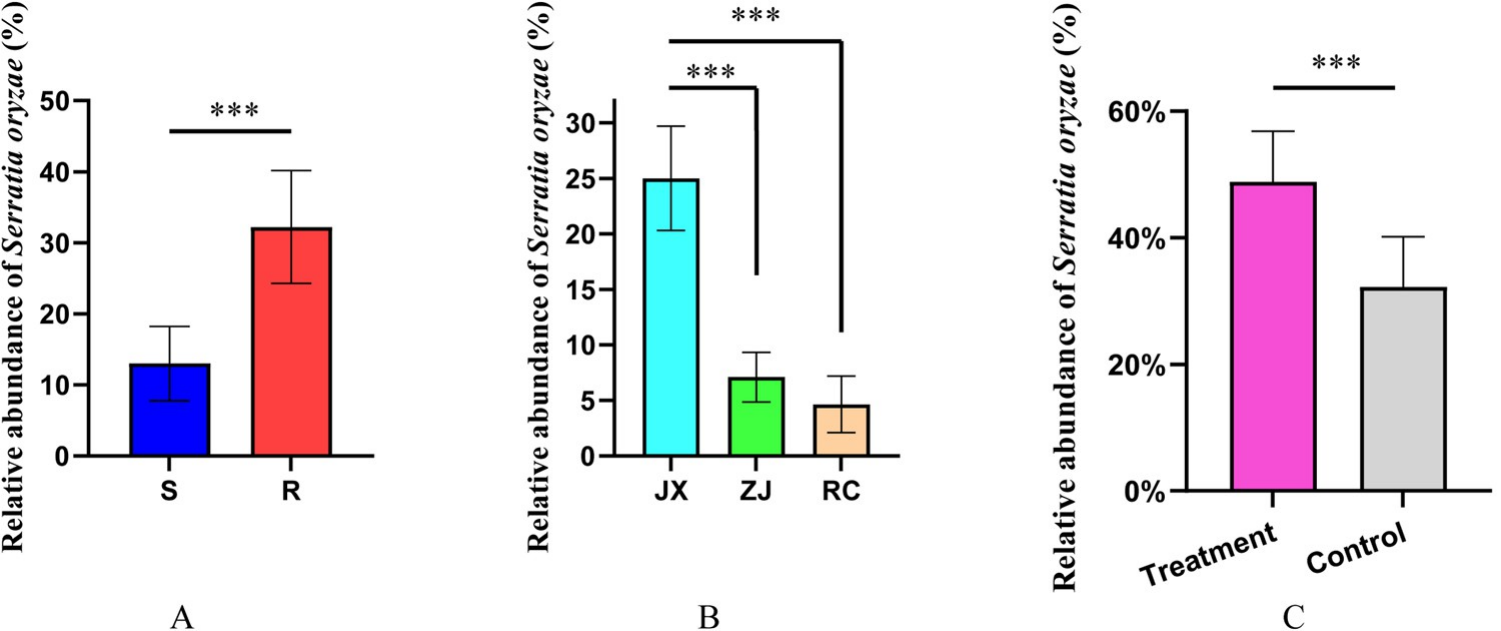
The relative abundance of *Serratia oryzae* of resistant mosquitoes significantly higher than that of sensitive mosquitoes (A); The relative abundance of *Serratia oryzae* of JX mosquitoes, that is field resistant mosquitoes, significantly higher than that of ZJ and RC mosquitoes (B); The relative abundance of *Serratia oryzae* of deltamethrin-treated mosquitoes significantly higher than the control groups (C).

Alpha diversity analysis was performed to examine the species diversity using Shannon (t = 8.26, df = 6, *p*<0.001) and Simpson (t = 8.26, df = 6, *p*<0.001) indices; also, the species abundance index, Chao1 (t = 3.811, df = 6, *p* = 0.009) was calculated (**Fig 1A–1C**). All the above indices suggested significant differences in symbiotic bacteria between laboratory strains that is resistant and susceptible mosquitoes. The symbiotic bacteria diversity and abundance were much higher in resistant mosquitoes than in susceptible mosquitoes. Interestingly, *S*. *oryzae* was the most abundant (32.24±7.94%) and significantly higher (t = 4.4, df = 7, *p* = 0.003) bacteria in resistant mosquitoes than in susceptible mosquitoes (**Fig 2A**). The resistant phenotypes among three field-caught mosquitoes showed variations; the LC_50_ of JX mosquitoes was higher than the ZJ and RC mosquitoes (**Table 1**). Also, the relative abundance of *S*. *oryzae* in JX mosquitoes was significantly higher than the ZJ (t = 7.654, df = 8, *p*<0.0001) and RC (t = 8.476, df = 8, *p*<0.0001) mosquitoes (**Fig 2B**). Notably, the relative abundance of *S*. *oryzae* also showed significant differences before and after insecticide treatment (t = 2.96, df = 6, *p* = 0.025) (**Fig 2C**).

### *S*. *oryzae* enrichment did not affect the normal activity of mosquitoes

**Fig 3:**
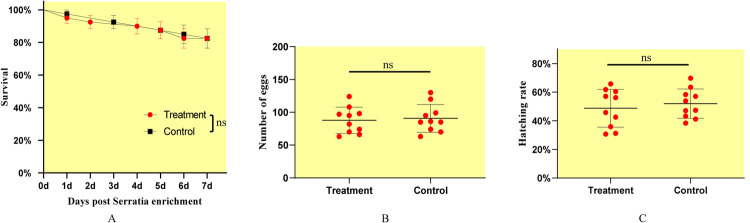
Treatment and control represent *Serratia oryzae*-enrichment and untreated mosquitoes, respectively. *Serratia oryzae*-enrichment in *Aedes albopictus*’ guts cannot affect the survival rate (A), number of eggs laid (B), and hatching rate (C).

Bacterial culture studies revealed that *S*. *oryzae* is a facultative anaerobe that grows as white to cream opaque colonies with raised and smooth surfaces, and neat edges on LB plate. The thallus showed fine rod shape, stained gram-negative, and have capsule without spore (**S3 Fig**). Survival studies revealed that in the first seven days, *S*. *oryzae*-enrichment did not influence the survival of mosquitoes (df = 1, *p* = 0.974) (**Fig 3A**). Also, the number of eggs from engorged-mosquito (t = 0.326, df = 18, *p* = 0.749) and hatching rate (t = 0.608, df = 18, *p* = 0.551) showed no difference between the bacteria enriched and control mosquitoes (**Fig 3B and 3C**). These results suggest that *S*. *oryzae* enrichment did not affect the normal life activities of mosquitoes and the conditions can be used for subsequent research.

### Generation of sterile and *S*. *oryzae*-enriched mosquitoes

Both the colony counting method and 16S rRNA gene absolute copy number quantitative method showed that gentamicin (150μg/mL) and streptomycin (150μg/mL) were effective against the intestinal symbiotic bacteria of mosquitoes (**S4A and S4B Fig**). Obviously, although we removed the bacteria in the midguts as much as possible, there were still some drug-resistant that could not be removed. Overall, we found that the samples obtained from this process can be used for subsequent experiments.

**Fig 4:**
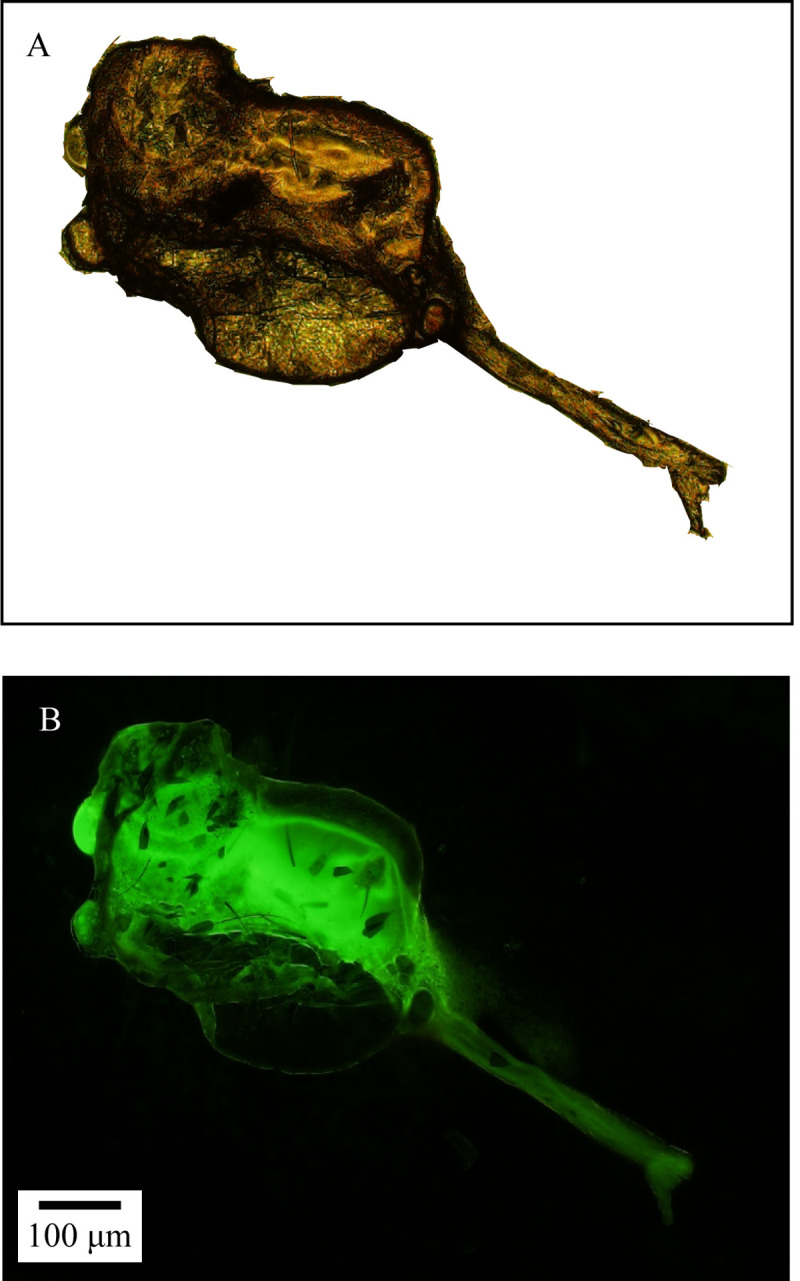
Visualization of GFP-tagged *Serratia oryzae* was successfully introduced into *Aedes albopictus*’ gut by feeding. Bright-field images (A) and the corresponding fluorescent images (B).

The plasmid expressing prokaryotic GFP was successfully introduced into the competent *S*. *oryzae* cells by the chemical transformation (CaCl_2_) method, and the transformed bacteria were successfully fed to the adult mosquitoes (**Fig 4**) for the intestinal enrichment of *S*. *oryzae*.

### *S*. *oryzae* enrichment enhances insecticide resistance

**Fig 5:**
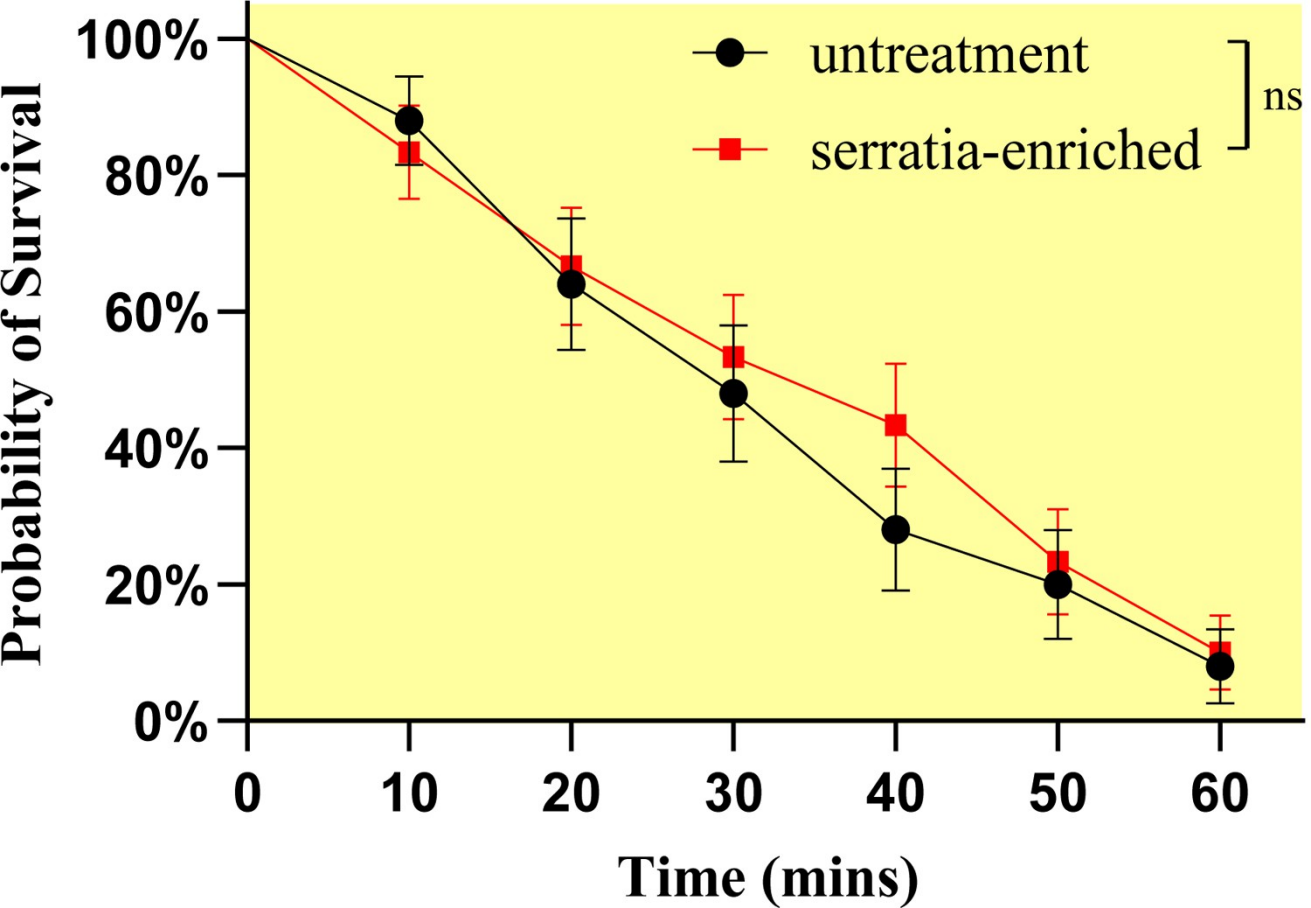
There was no significant difference in knockdown rate between the *Serratia oryzae* enrichment and control groups within 60mins.

There was no significant difference in knockdown rate between the *S*. *oryzae* enrichment and control groups (df = 1, *p* = 0.6103) (**Fig 5**) within 60 min; however, the survival rate of the *S*.*oryzae* enrichment group was significantly higher than the control group after 24 h recovery (χ^2^ = 4.583, df = 1, *p* = 0.032), indicating that *S*. *oryzae* promoted insecticide resistance. The CDC bottle knockdown experiment was carried out on 3 field strains, and 2 laboratory strains *i*.*e*, resistant and susceptible strains. The survival rate (**Table 2**) after 24 h recovery showed a positive correlation with the relative abundance of *S*. *oryzae* in female mosquitoes (r^2^ = 0.953, *p* = 0.012), indicating the *S*. *oryzae* effect on resistance enhancement.

**Table 2:** The average abundance of *Serratia oryzae* and the survival rate of five strains of mosquitoes.

	S	R	ZJ	RC	JX
Mean abundance (%)	12.96	32.24	7.10	4.65	24.96
Survival rate (%)	11.11	48.28	6.45	13.33	40.0

The CDC bottle knockdown experiment was carried out on five strains of mosquitoes. The survival rate refers to the proportion of female mosquitoes that can fly normally after 24h recovery. The mean abundance means the relative abundance of *Serratia oryzae* in intestinal symbiotic bacteria.

### *S*. *oryzae* promotes the activity of three insecticide metabolic enzymes

**Fig 6:**
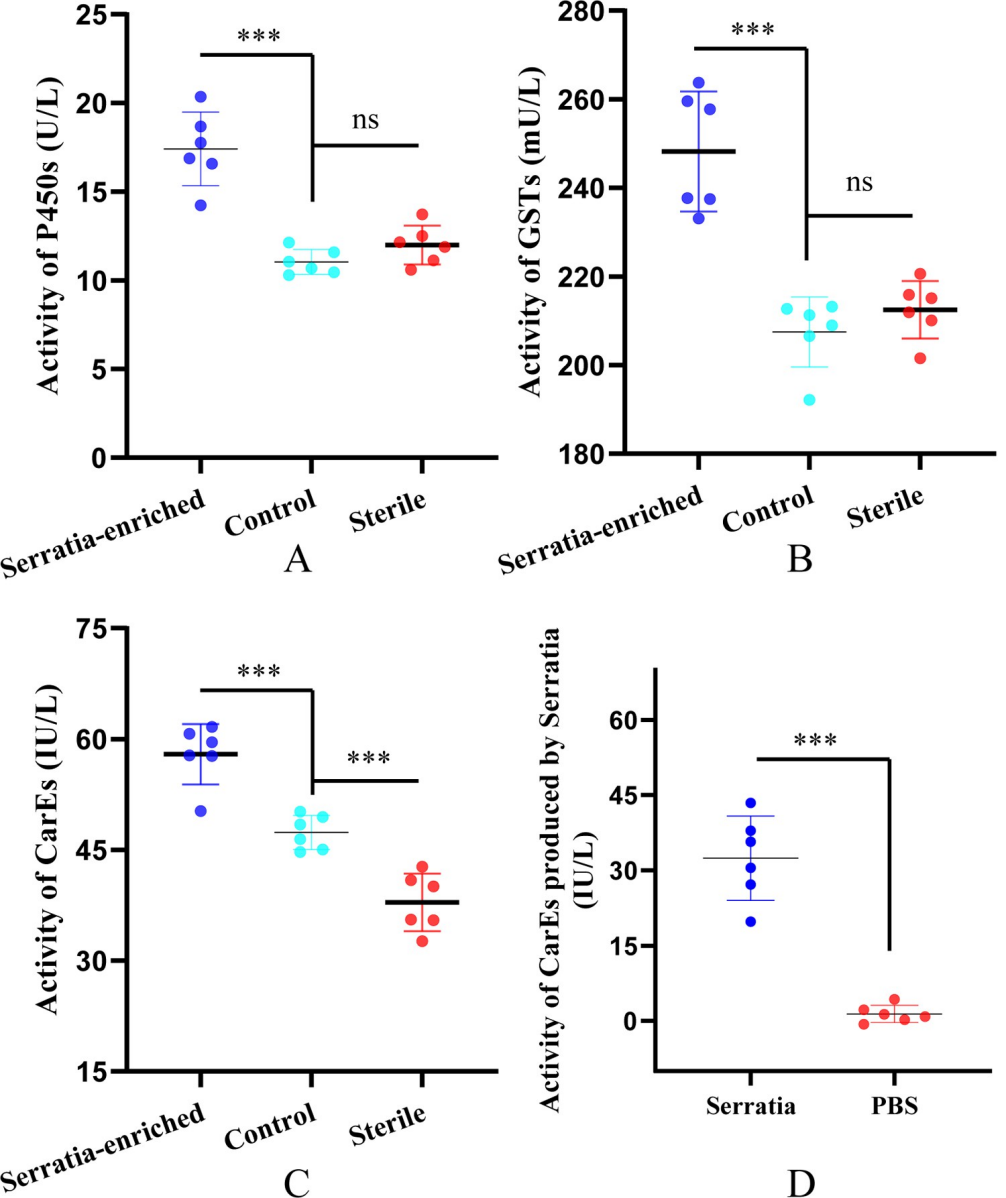
Visualization of activity comparison of P450s (A), GSTs (B), CarEs (C) between *Serratia oryzae*-enrichment, sterile mosquitoes and untreated groups (control). D shows the activity of CarEs of produced by *Serratia oryzae*.

We tested the activities of P450s, GSTs, and CarEs. Compared with the control group, the activities of P450s (t = 7.134, df = 10, *p*<0.001), GSTs (t = 6.368, df = 10, *p*<0.001), and CarEs (t = 5.517, df = 10, *p*<0.001) were significantly higher in *S*. *oryzae*-enriched mosquitoes. This indicated *S*. *oryzae* inducing effect on the activities of these three enzymes. However, when the midgut bacteria were removed by antibiotics, only CarEs activity declined (t = 5.129, df = 10, *p*<0.001). Moreover, we detected the extracellular enzymes of *S*. *oryzae*, but only CarEs activity was found in the culture supernatant (t = 8.849, df = 10, *p*<0.001) (**Fig 6**), indicating *S*. *oryzae* secretes CarEs *in vitro*.

### Validation of DEGs

**Fig 7:**
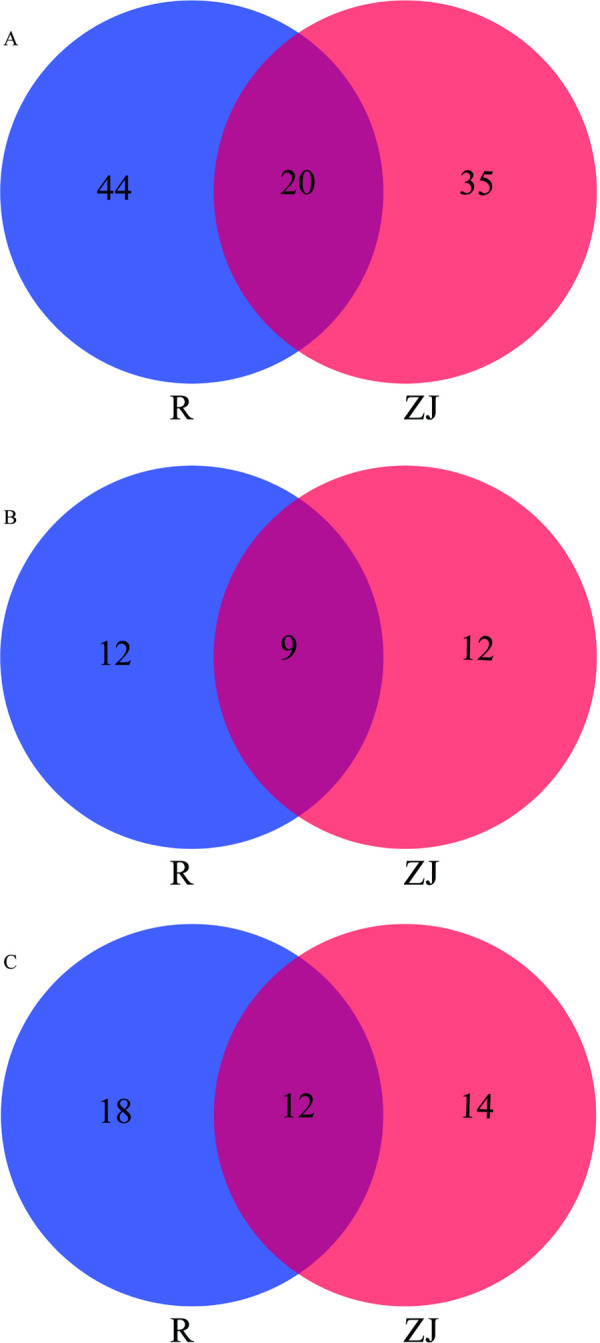
Co-upregulated gene number of P450s (A), GSTs (B) and CarEs (C) genes, based on RNA-Seq, after *Serratia oryzae* enriched. The intersections represent the number of up-regulated genes after bacterial enrichment in both field (ZJ mosquitoes) and laboratory (R mosquitoes) strains.

The transcriptomic data of metabolic detoxification enzyme genes in laboratory and field strains before and after *S*. *oryzae* enrichment are shown in **Fig 7**. The cDNA of mosquitoes before and after *S*. *oryzae* enrichment was used as the template, and the DEGs were validated by RT-qPCR. The relative differential expression multiples of 9 P450s, 8 GSTs, and 7 CarEs genes were consistent with the transcriptional results (**S2 Table**).

### Mode of *S*. *oryzae* transmission and its ability to degrade insecticides *in vitro*

**Fig 8:**
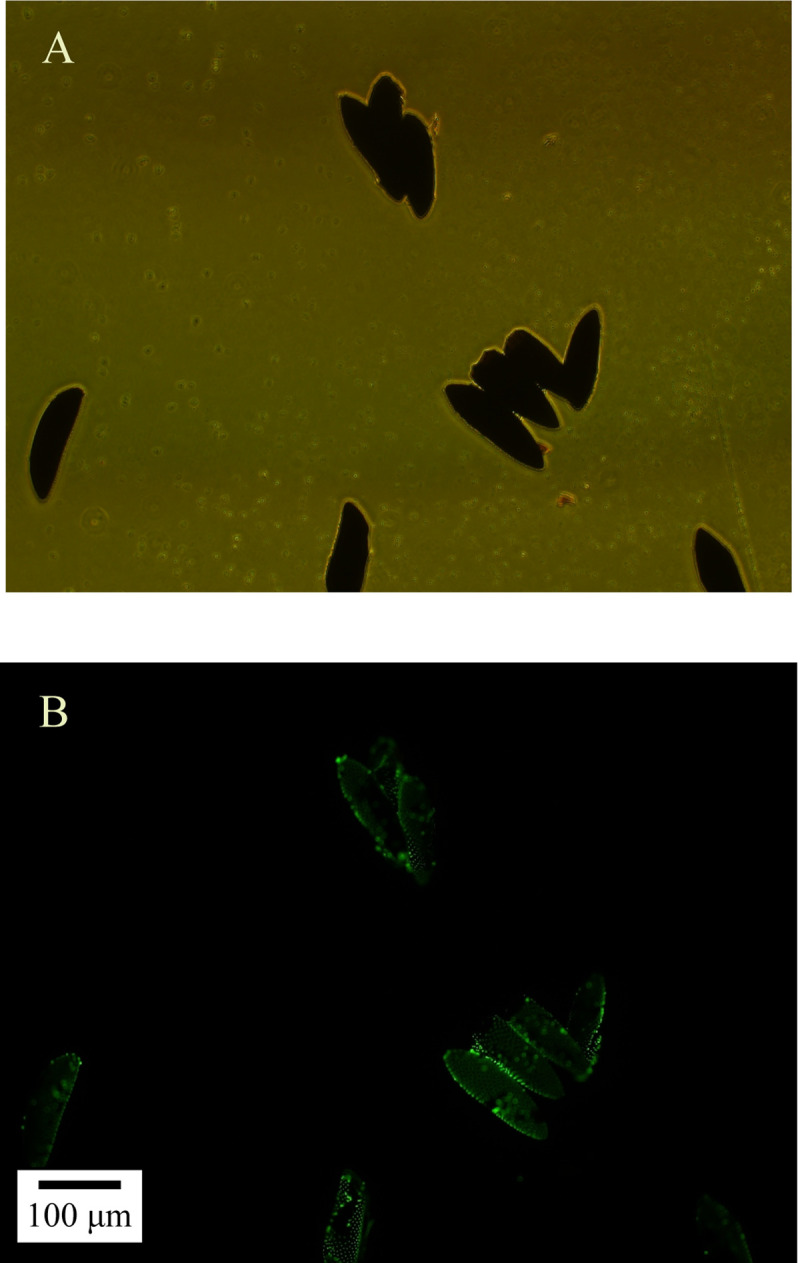
Visualization of *Serratia oryzae* can be transmitted by female *Aedes albopictus*. Bright-field images (A) and the corresponding fluorescent images (B).

The eggs laid by GFP-Tagged *S*. *oryzae* fed female mosquitoes were collected. These showed green fluorescent substances under the fluorescence microscope (**Fig 8**), indicating that *S*. *oryzae* can be transmitted via vertical propagation. In addition, the larvae and water collected from the breeding sites were analyzed for bacterial species. We found that *S*. *oryzae* was present only in the larvae’s midguts, but not in water (**S5 Fig**). This further indicates that mosquitoes acquired *S*. *oryzae* from their parental generation and not from the breeding sites.

**Table 3** shows the average residual concentration of deltamethrin in each group of media at different culture duration. We established the logistic multiple linear regression model with the average residual concentration as the dependent variable and the culture time and bacterial concentration as the independent variables. The regression equation: Y = 0.152–0.013 (Time) -0.014 (Concentration), R^2^ = 0.736, F = 27.506, *p*<0.0001 indicated that *S*. *oryzae* could degrade deltamethrin *in vitro*, and the degradation was dependent on degradation time and bacterial amount. Moreover, the results of GC-MS/MS studies show that deltamethrin can be degraded into 1-Oleoyl-2-hydroxy-sn-glycero-3-PE and 2’,2’-Dibromo-2’-deoxyguanosine by the hydration, palmitoyl conjugation, oxidation, and taurine conjugation reactions (**Table 4**).

**Table 3:** The average residual concentration of deltamethrin in each group of media at different culture duration.

	OD_600_ = 0.5 (mg/L)	OD_600_ = 1.0 (mg/L)	OD_600_ = 2.0 (mg/L)	Control (mg/L)
initial concentration	0.1	0.1	0.1	0.1
12 h	0.097	0.096	0.087	0.1
24 h	0.095	0.076	0.046	0.1
36 h	0.086	0.057	0.029	0.098
48 h	0.057	0.024	0.023	0.098

Three groups of media contained different concentration *Serratia oryzae*, that is OD_600_ = 0.5, 1.0 and 2.0. The control group contained the same concentration of deltamethrin without *Serratia oryzae*. The residual concentration of deltamethrin (mg/L) at different time point was collected and recorded in the table.

**Table 4:** Results of substance metabolism and full-spectrum comparison.

Parent Compound	Dealkylated	Transformations	Composition Change	Formula	Calc. MW	Name	Area
Deltamethrin	TRUE	Oxidation, Palmitoyl Conjugation	-(Br_2_)+(C_8_H_22_O)	C_30_H_41_NO_4_	479.301	1-Oleoyl-2-hydroxy-sn-glycero-3-PE	5200840.05
Deltamethrin	TRUE	Hydration, Palmitoyl Conjugation	-(Br_2_)+(C_8_H_22_O)	C_30_H_41_NO_4_	479.301	1-Oleoyl-2-hydroxy-sn-glycero-3-PE	5200840.05
Deltamethrin	TRUE	Hydration, Taurine Conjugation	-(C_12_H_2_)+(O_2_S)	C_10_H_17_Br_2_N O_5_S	422.915	2’,2’-Dibromo-2’-deoxyguanosine	891096.5151

Dealkylated means whether dealkylation; Transformations means rection type; Composition Change means a change in the chemical formula; Formula means the molecular formula of the transformed compound; Calc. MW means the molecular weight of the transformed compound; Name means the full-spectrum identification of compound names. Area means peak area detected.

## Discussion

Owing to high insecticide efficacy and non-toxicity towards humans and other vertebrates, pyrethroids are the most commonly used insecticide to resist mosquitoes [34,35]. However, due to increased agricultural production and population flow which contributed to excessive reliance on pesticides in Shandong province in the past 20 years, the local mosquitoes are generating strong resistance against it, especially *Ae*. *albopictus* and *C*. *p*. *pallens* [36,37]. Therefore, understanding the mechanism of insecticide resistance in mosquitoes is necessary to develop new mosquitoes control strategies. Many current studies have focused on the relationship between intestinal symbiotic bacteria and insecticides resistance. It is widely accepted that insect intestinal symbiotic bacteria are involved in host resistance to insecticides, at least in agricultural insects. For example, Citrobacter in the intestinal tract of *Bactrocera dorsalis* (Diptera) increases host resistance to trichlorfon by improving its degradation [38]. Likewise, Burkholderia increases *Riptortus pedestris* (Hemiptera) resistance to fenitrothion [39]. In mosquitoes, it is reported that symbiotic bacteria can promote the process of metabolic detoxification via their own and host enzyme systems. Symbiotic bacteria can use compounds such as insecticides to transfer carbon source into energy for their growth [40]. Werren et al. showed that bacteria can metabolize insecticides and use them as sources of carbon, phosphorus, or nitrogen, facilitating their degradation, at least, *in vitro* [41]. Also, some studies showed that the increased bacterial diversity and abundance in mosquito’s guts enhances hosts’ metabolic kinetics promoting insecticides resistance [42]. Under the selection pressure of the intestinal environment, symbiotic bacteria have evolved highly efficient enzymes from a wide variety of enzyme families [40]. Apart from biodegradation, symbiotic bacteria can also regulate the host immune response against insecticides by inducing the expression of immune-related genes. However, such pathways tend to be used for resisting the biological insecticides, such as *Metarhizium anisopliae*, *Beauveria bassiana*, and *Bacillus thuringiensis* [43]. All these studies suggest that symbiotic bacteria can increase mosquito’s resistance to insecticides.

*S*. *oryzae*, initially isolated from rice stems [44], was also isolated from lake water [31]. It is known to produce extracellular cellulase and protease. The first mosquito found to carry *S*. *oryzae* was *An*. *arabiensis* [45]. This research firstly proved the effect of *S*. *oryzae* on *Ae*. *albopictus* resistance to insecticide by screening five different strains of mosquitoes. Notably, *S*. *oryzae* is a dominant bacterium in both male and female *An*. *arabiensis* and *An*. *funestus* mosquitoes, indicating *S*. *oryzae*’s role in both species [45].

For the mechanistic understanding of the resistance, we constructed *S*. *oryzae* expressing GFP and introduced it into adult mosquitoes by artificial feeding method. This not only proved that the bacteria can be introduced into adult mosquitoes by artificial feeding but also established a model to study the effect of introduced bacteria on the normal physiological activities and life cycle of *Ae*. *albopictus* [46]. To study the mechanism of *S*. *oryzae* induced resistance, female susceptible mosquitoes were treated with sterilized and increasing concentrations of bacteria, and then changes in the metabolic detoxification enzyme activity were estimated. Although we used a mixture of gentamicin (150μg/mL) and streptomycin (150μg/mL) to remove (kill) the intestinal symbiotic bacteria, the complete removal of all bacteria is impossible in the case of some drug-resistant bacteria [19]. The results showed that among the three metabolic detoxification enzymes, only the esterase activity decreased significantly in the sterile mosquitoes. This suggests that the presence of esterase secreting bacteria in the intestinal tract. In addition, the activities of three major metabolic detoxification enzymes were significantly higher in *S*. *oryzae*-enriched mosquitoes; notably, *S*. *oryzae* itself does not secrete P450s and GSTs. Therefore, we next examined the changes in gene expression of metabolic detoxification enzymes before and after bacterial enrichment and found that the gene expression levels of the three metabolic detoxification enzymes were up-regulated to varying degrees in *S*. *oryzae* enriched mosquitoes, suggesting *S*. *oryzae* might enhance the host metabolic detoxification ability. Among the up-regulated genes, the insecticides metabolic activity of CYP6, CYP9, GSTE4, and GSTE6 families of enzymes is widely known [47–49]. Also, *S*. *oryzae* could secrete carboxylesterase *in vitro*, which could have promoted deltamethrin resistance of the host. The same has been confirmed for *Enterococcus sp*. (Firmicutes) too [50]. In addition, some studies showed that apart from biodegradation activity, intestinal bacteria may improve insecticide resistance by protecting the immune system of insect hosts [51,52]. However, due to limited resources, we could not focus on the immune system in this study.

We demonstrated that *S*. *oryzae* can degrade deltamethrin into 1-Oleoyl-2-hydroxy-sn-glycero-3-PE and 2’,2’-Dibromo-2’-deoxyguanosine through a series of biochemical reactions including hydration, palmitoyl conjugation, oxidation, and taurine conjugation reactions *in vitro*. This process is concentration- and time-dependent, *i*.*e*. with the increase of bacterial abundance and the prolongation of reaction time, the degradation effect became more significant. This also means that this may be a slow biological process, which explains why there was no difference in knockdown rate, but the survival rate became prominent after 24h of recovery. We did not study *S*. *oryzae*’s ability to degrade other types of insecticides, such as propoxur due to its strong cytotoxicity causing oxidative damage to mammals [53]. One of the most significant targets of oxidative damage is DNA that can promote apoptosis [54]. In addition, it causes acute carbamate compound poisoning in humans and other mammals by inhibiting acetylcholinesterase [55]. Therefore, these insecticides are often used in agricultural production, and less often used in mosquito control.

The transmission of *S*. *oryzae* in *Ae*. *albopictus* population is an important event. We found that *S*. *oryzae* was absent in *Ae*. *albopictus* breeding water bodies such as small water containers, lotus ponds, and small puddles. However, *S*. *oryzae* was detected in larvae captured from the breeding sites (**S2 Fig**). This suggests that water bodies were not the source of *S*. *oryzae* enrichment in *Ae*. *albopictus*. Researchers used MALDI-TOF MS to show that although *S*. *oryzae* was abundant in the intestinal tract of *An*. *arabiensis*, it was absent in its small aquatic habitat [45]. This is consistent with our finding that mosquitoes cannot directly acquire the bacteria from their habitat water bodies. Also, the eggs laid by GFP-tagged *S*. *oryzae*-enriched *Ae*. *albopictus* showed green fluorescence (**Fig 8**). Therefore, these are good reasons to suggest that *S*. *oryzae* spread in the population through vertical propagation and not through the breeding sites. Without affecting the hosts, this kind of bacteria proliferation begins under insecticide selection pressure owing to their ability to degrade insecticides and use them as a nutrition source. However, as to how *Ae*. *albopictus* initial *S*. *oryzae*, it requires species tracing.

Given the *S*. *oryzae*’s ability to degrade deltamethrin *in vitro*, in combination with the damage of pesticide residue to cultivated land and water environment because of the overdependent on insecticides, we speculate that if further studies on the bacterial degradation of pesticides, such as targets and function way, and to participate in the main protein degradation. It not only elucidates the latest mechanism of resistance of *Ae*. *albopictus*, but also may accelerates the commercial use of the bacterium to degrade pesticide residues in farmland and irrigated lakes, thus protecting the environment and ecology [56,57].

In conclusion, this work demonstrates that intestinal symbiotic bacteria participate in the promotion of *Ae*. *albopictus* resistance, which strongly complements the resistance research in *Ae*. *albopictus*. This work is of great significance for the management of mosquito resistance and the prevention and control of mosquito-borne diseases. However, this study also has some limitations, such as not being able to identify how the *S*. *oryzae* regulate the expression of detoxification genes, and whether it can affect the transmission efficiency of *Ae*. *albopictus* to vector viruses by participating in the regulation of host resistance.

## Conclusions

Being a symbiotic and stable parasitic bacterium, *S*. *oryzae* can be accumulated into adult *Ae*. *albopictus’* midgut by artificial feeding, which enhances its deltamethrin resistance. In the wild, it can vertically transmit among *Ae*. *albopictus* population and thrive in midguts under the insecticide selection pressure. As the number of bacteria accumulates, it can significantly enhance the deltamethrin resistance by up-regulating the expression of metabolic detoxification genes. Importantly, *S*. *oryzae* can use deltamethrin as the sole carbon source, indicating its ability to degrade deltamethrin *in vitro*. Therefore, in the future, *S*. *oryzae* may also be commercially used to break down the residual insecticides in the farmland and lakes to protect the environment.

## Supporting information

S1 Fig CDC bottle bioassay indicator diagram. The left figure shows the treatment groups, and the right figure shows the restored groups after 1 h. Both of them show the comparison of knock down rate between *Serratia oryzae-*treatment and untreatment groups, and the comparison of survival rate after recovering 24 h.
(TIF)

S2 Fig The eggs laid by GFP-Tagged *Serratia oryzae* fed female mosquitoes were collected, and the green fluorescent substances was found under the fluorescence microscope (A). *Serratia oryzae* was found in the larvae’s midguts, but not in breeding sites (B).
(TIF)

S3 Fig The gram straining (A), spore straining (B), and capsule straining (C) were observed under microscope (1000×). The red arrows (C) indicate *Serratia oryzae*’s capsule.
(TIF)

S4 Fig Both the results of colony-counting method (A) and 16S rRNA gene copy number method (B) indicate that antibiotic treatment is effective.
(TIF)

S5 Fig *Serratia oryzae* colonize in the intestinal symbiotic bacteria of three strains of larvae (A), but not in the water sample (B) collected from their breeding sites.
(TIF)

S1 Table Primer list. (DOCX)

S2 Table RNA-seq results validated by RT-qPCR. (DOCX)
