# Supplementary figures and images for: A symbiotic gut bacterium enhances Aedes albopictus resistance to insecticide
Source: PLoS Negl Trop Dis. 2022 Mar 4;16(3):e0010208. doi: 10.1371/journal.pntd.0010208 (PMC8896681; doi:10.1371/journal.pntd.0010208)

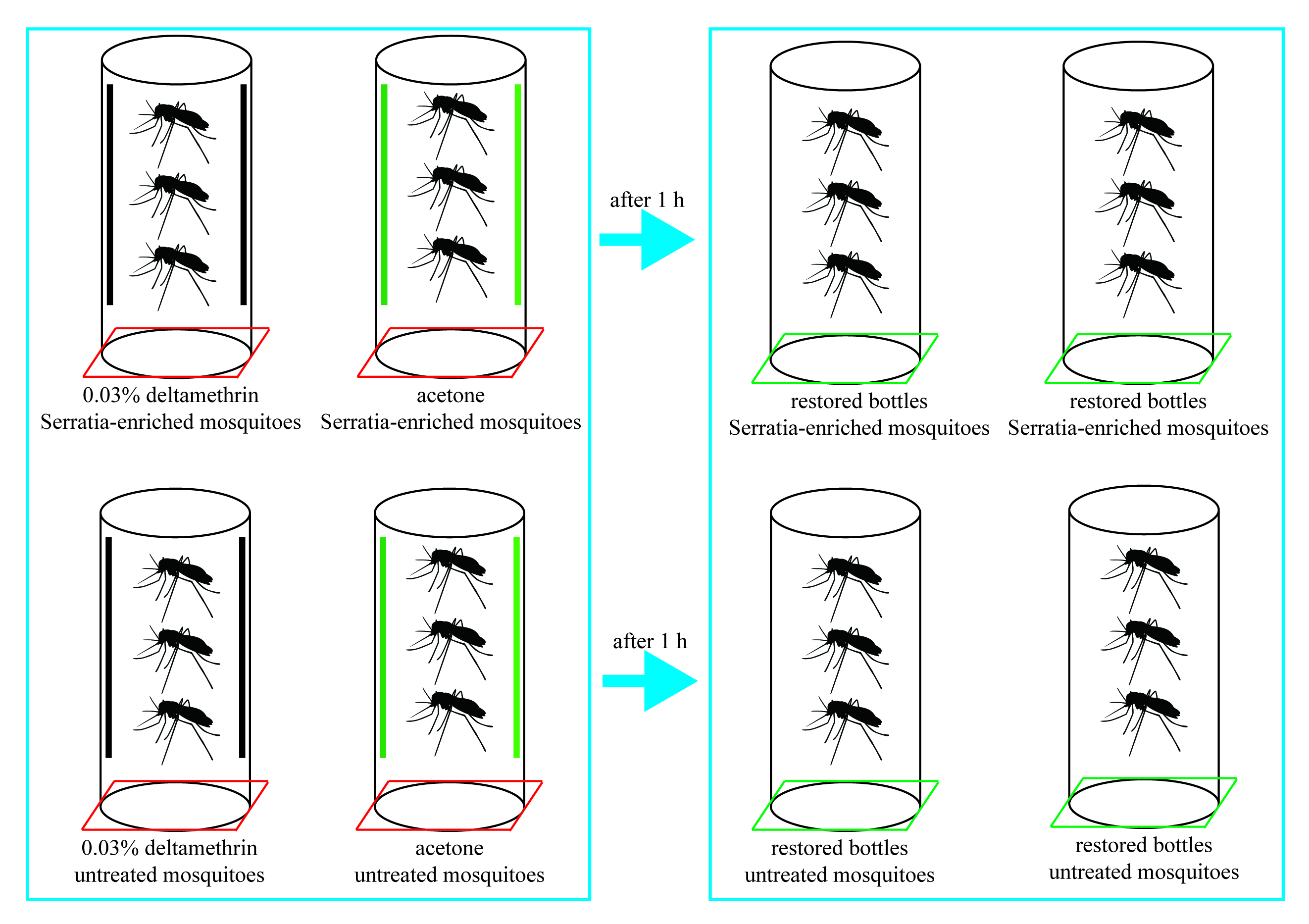

Supplement: S1 Fig — The left figure shows the treatment groups, and the right figure shows the restored groups after 1 h. Both of them show the comparison of knock down rate between Serratia oryzae-treatment and untreatment groups, and the comparison of survival rate after recovering 24 h. (TIF) [file pntd.0010208.s001.tif]

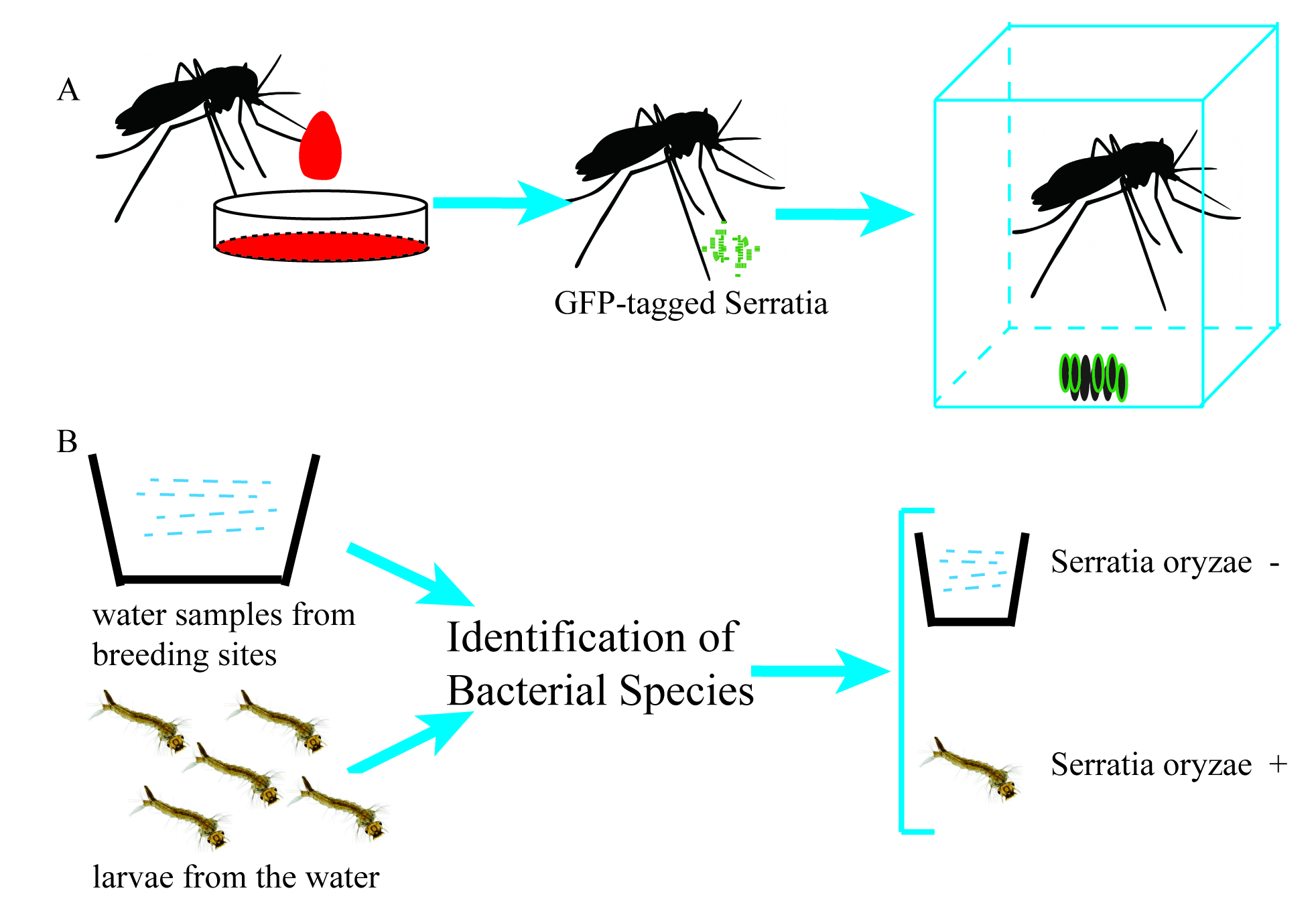

Supplement: S2 Fig — The eggs laid by GFP-Tagged Serratia oryzae fed female mosquitoes were collected, and the green fluorescent substances was found under the fluorescence microscope (A). Serratia oryzae was found in the larvae’s midguts, but not in breeding sites (B). (TIF) [file pntd.0010208.s002.tif]

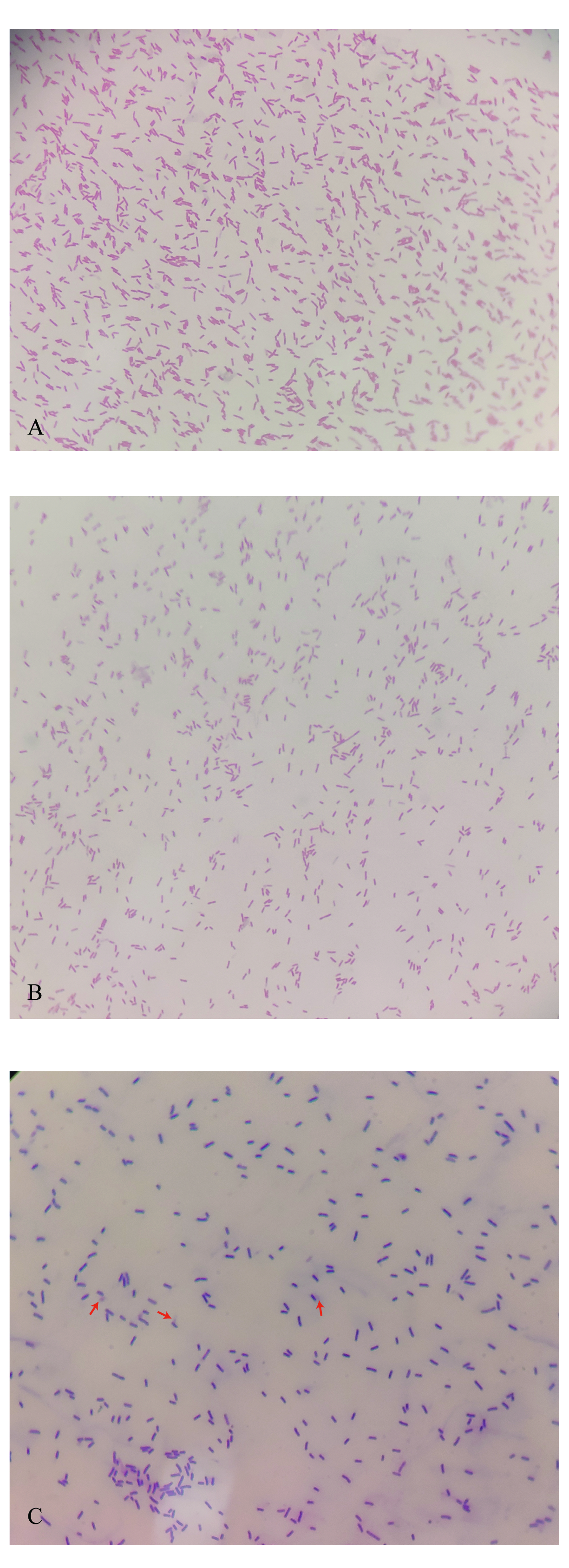

Supplement: S3 Fig — The gram straining (A), spore straining (B), and capsule straining (C) were observed under microscope (1000×). The red arrows (C) indicate Serratia oryzae’s capsule. (TIF) [file pntd.0010208.s003.tif]

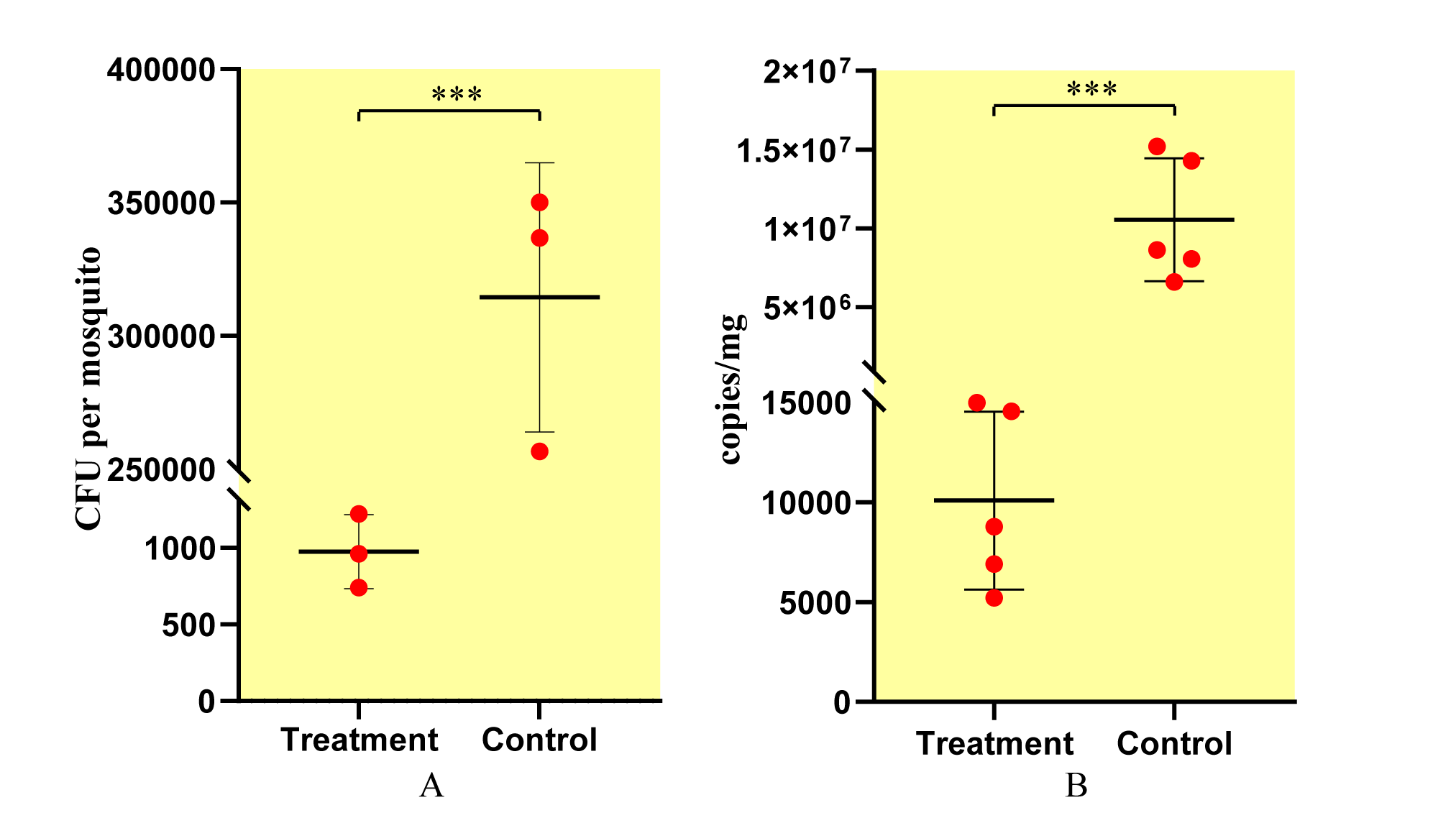

Supplement: S4 Fig — Both the results of colony-counting method (A) and 16S rRNA gene copy number method (B) indicate that antibiotic treatment is effective. (TIF) [file pntd.0010208.s004.tif]

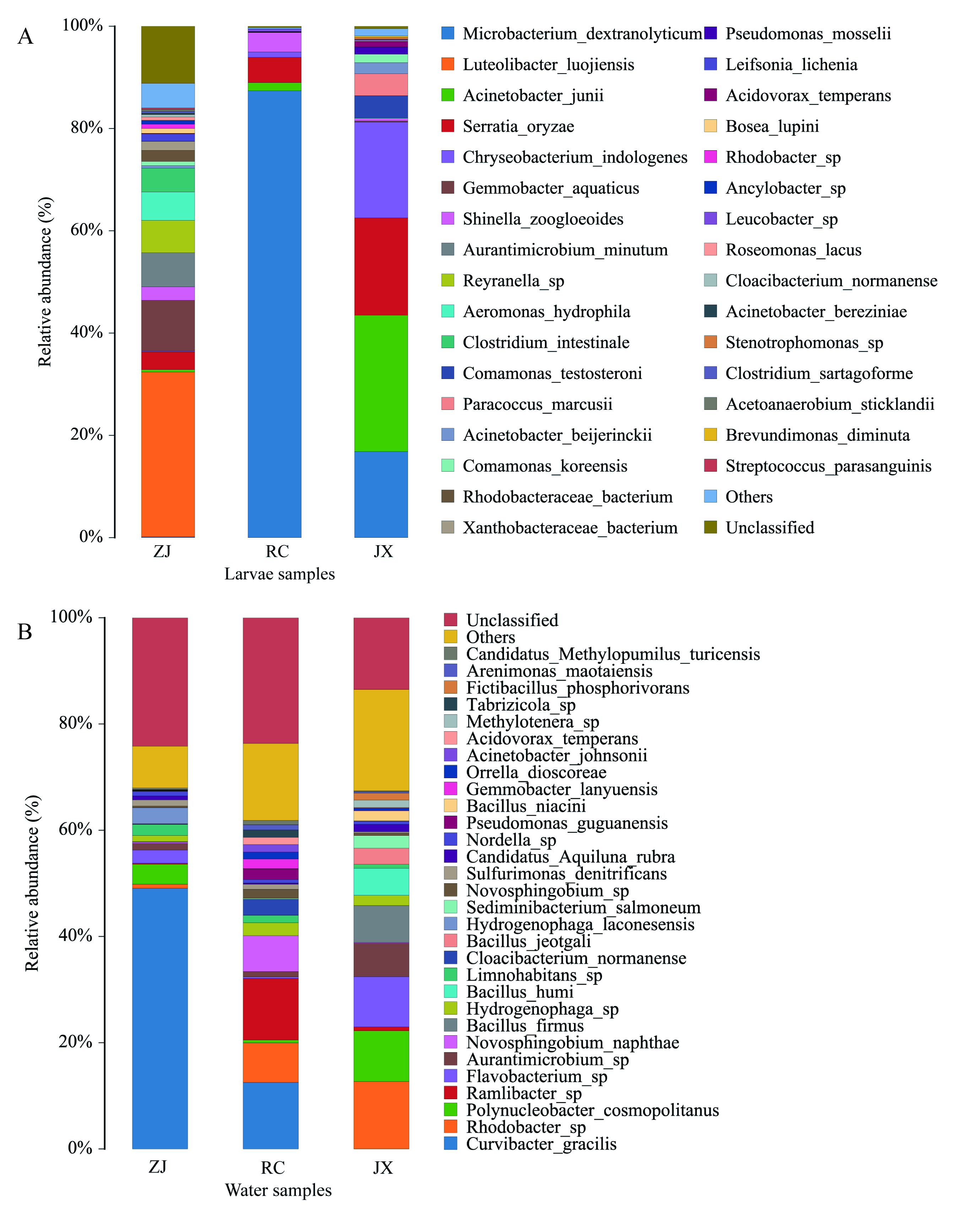

Supplement: S5 Fig — Serratia oryzae colonize in the intestinal symbiotic bacteria of three strains of larvae (A), but not in the water sample (B) collected from their breeding sites. (TIF) [file pntd.0010208.s005.tif]
