# Supplementary material for: A symbiotic gut bacterium enhances Aedes albopictus resistance to insecticide
Source: PLoS Negl Trop Dis. 2022 Mar 4;16(3):e0010208. doi: 10.1371/journal.pntd.0010208 (PMC8896681; doi:10.1371/journal.pntd.0010208)
Supplement: S1 Table — (DOCX) [file pntd.0010208.s006.docx]

**S1 Table** Primer list

| Target gene name or ID | Primer name | Sequence |
| --- | --- | --- |
| full-length of 16S rRNA | 27F | AGRGTTTGATYNTGGCTCAG |
|  | 1492R | TASGGHTACCTTGTTASGACTT |
| V4-V5 of 16S rRNA | 515F | GTGCCAGCMGCCGCGG |
|  | 907R | CCGTCAATTCMTTTRAGTTT |
| AALF006036 | F | GTTGAGGAGTCTGAAGTCGGAAAGTC |
|  | R | CTGCCAGGAAGAATACCAAACATTGC |
| AALF005550 | F | ATGTGCCTTTCCTGAAACCGTATCC |
|  | R | CAGGCTCACGGAACGAGAAGAATC |
| AALF000820 | F | CGCTACGCCAACAAGGACTACATC |
|  | R | AGTGCCAGCATCGGAATCCAAAC |
| AALF016136 | F | TGGAGATCAAGGCAATCATGTACGC |
|  | R | TTAACCAACCTCAACGGCACACTAG |
| AALF023150 | F | ATTCGTATGTGACACTGATGGCTCTG |
|  | R | TAACAACTCGCAACAGGAATAGGACAG |
| AALF015441 | F | GCCATTCGCTACATCTACTCGTACTG |
|  | R | TTTCAGGTTGCCGTAAGGGATATGC |
| AALF017104 | F | CAGTGGCAAATTCGTGGACTTTATCG |
|  | R | CGGCAACATTCTGGGTCGTATAGG |
| AALF007271 | F | AGACCTCTAGTGCCACCCTTTCC |
|  | R | TCGTTGGTTGCGTTGAGGAGTTC |
| AALF012355 | F | TGATACTGCTGTTGCTGCTGGTTG |
|  | R | AGCGGATACGGTTTCAAGAAAGGAAC |
| AALF001852 | F | ATGGCGACTTCTCTTTGAACGATCC |
|  | R | TCTCTCTGAAACAGCACGGCATTG |
| AALF018084 | F | TCAGTTGCGTGACTTCGTTCTCG |
|  | R | TGATCTCGTCGTACCCCTGGATG |
| AALF018085 | F | ACGATAGCCATGCCATTATCATCTACC |
|  | R | CGAGCGAACAGAACTCCCGAATC |
| AALF021145 | F | TACCTGGAGAAGCTGGACGACATC |
|  | R | GGTCCGACTTGGTCATGTAGTTCAG |
| new gene23301 | F | GCCATCATCATCTACCTAGTGAGCAAG |
|  | R | CGAGCGAACAGAACTCCCGAATC |
| AALF001261 | F | TAGCAACGTGATCCAACCCTAAAGC |
|  | R | TGACAGCAGATCGTAGAAGAACTTGAC |
| AALF016463 | F | GGCAGCACGACAACAGTTCAAAG |
|  | R | CCCGTTCCAGTTGGTTCAGGTTC |
| AALF018090 | F | TTCGATGCCGTTCTGCCAATCAG |
|  | R | CGTTACCGTTCGCCTCCTTATAGTATG |
| AALF004602 | F | ACTGAGATGGTACAGAAGGCTAGAGAC |
|  | R | CCGACACATTCTTCAACACTTGTAACG |
| AALF009268 | F | ATTCTCACGCAATCCGCAATTCTTTG |
|  | R | TTGGCTGACCGCACTTGATTCTG |
| AALF009269 | F | CACCATTTCACCACGGGTTTCTTTATC |
|  | R | ATTACTACCTTACCTGCGAACGAAGC |
| AALF009271 | F | AACACAGCAGCAACATACTACTACCAG |
|  | R | GCCAGTCGCTAAGCAGAAGAAGG |
| AALF023063 | F | ACTGAGATGGTACAGAAGGCTAGAGAC |
|  | R | CCGACACATTCTTCAACACTTGTAACG |
| new Gene5082 | F | ATCAAGAGCCAACCTTCCTTACTCAAC |
|  | R | ACTTTCAGCACTCGTGTAGCCAATC |
| new Gene5083 | F | GGCAGCATACGGTTCCCTTTCTC |
|  | R | AACGGCACGAACAGATCAGGATTG |
| β-actin | F | GCCGTCTTCCCGTCCAT |
|  | R | GGCGACACGCAGCTCATT |

The letters F and R refer to the forward primer and reverse primer, respectively. β-actin is the reference gene.
