# Supplementary material for: A symbiotic gut bacterium enhances Aedes albopictus resistance to insecticide
Source: PLoS Negl Trop Dis. 2022 Mar 4;16(3):e0010208. doi: 10.1371/journal.pntd.0010208 (PMC8896681; doi:10.1371/journal.pntd.0010208)
Supplement: S2 Table — (DOCX) [file pntd.0010208.s007.docx]

**S2 Table** RNA-seq results validated by RT-qPCR

| P450s | | | | GSTs | | | | CarEs | | | |
| --- | --- | --- | --- | --- | --- | --- | --- | --- | --- | --- | --- |
| gene ID | gene name | log2FC | 2^^ΔΔCt^ | gene ID | gene name | log2FC | 2^^ΔΔCt^ | gene ID | gene name | log2FC | 2^^ΔΔCt^ |
| AALF000820 | CYP9J2 | 2.39 | 5.07 | AALF001852 | GSTE8 | 2.37 | 2.53 | AALF004602 | CCEAE1O | 2.41 | 10.25 |
| AALF005550 | CYP9M9 | 1.58 | 2.61 | AALF018084 | GSTE1 | 2.62 | 1.94 | AALF009268 | CCEAE3C | 4.02 | 12.06 |
| AALF006036 | CYP9J22 | 1.90 | 4.97 | AALF018085 | GSTE4 | 2.74 | 1.17 | AALF009269 | CCEAE4 | 3.80 | 11.35 |
| AALF012355 | CYP9E2 | 1.94 | 2.24 | AALF021145 | GSTS1 | 1.88 | 1.50 | AALF009271 | CCEAE5C | 2.14 | 13.01 |
| AALF015441 | CYP6Z8 | 1.34 | 1.01 | new gene23301 | GSTE4 | 3.18 | 1.19 | AALF023063 | ESTB1 | 2.09 | 9.88 |
| AALF016136 | CYP9F2 | 2.18 | 4.09 | AALF001261 | GSTT4 | 1.32 | 2.39 | new Gene5082 | CCEAE1C | 5.97 | 11.77 |
| AALF017104 | CYP6A13 | 1.72 | 1.21 | AALF016463 | GSTT4 | 1.21 | 2.88 | new Gene5083 | CCEAE6C | 1.92 | 4.66 |
| AALF023150 | CYP4D14 | 2.82 | 4.86 | AALF018090 | GSTE6 | 1.87 | 1.37 |  |  |  |  |
| AALF007271 | CYP28D1 | 2.25 | 4.98 |  |  |  |  |  |  |  |  |

There are 9 P450s, 8 GSTs, and 7 CarEs genes listed in this table, whose quantitative results of qPCR were consistent with the transcriptional results. 2^^ΔΔCt^ refers to the differential expression multiple.
